# Pseudospin-1 Physics of Photonic Crystals

**DOI:** 10.34133/2019/3054062

**Published:** 2019-04-08

**Authors:** A. Fang, Z. Q. Zhang, Steven G. Louie, C. T. Chan

**Affiliations:** ^1^Department of Physics, The Hong Kong University of Science and Technology, Clear Water Bay, Hong Kong, China; ^2^Institute for Advanced Study, The Hong Kong University of Science and Technology, Clear Water Bay, Hong Kong, China; ^3^Department of Physics, University of California at Berkeley, Berkeley, CA 94720, USA; ^4^Materials Sciences Division, Lawrence Berkeley National Laboratory, Berkeley, CA 94720, USA

## Abstract

We review some recent progress in the exploration of pseudospin-1 physics using dielectric photonic crystals (PCs). We show some physical implications of the PCs exhibiting an accidental degeneracy induced conical dispersion at the Γ point, such as the realization of zero refractive index medium and the zero Berry phase of a loop around the nodal point. The photonic states of such PCs near the Dirac-like point can be described by an effective spin-orbit Hamiltonian of pseudospin-1. The wave propagation in the positive, negative, and zero index media can be unified within a framework of pseudospin-1 description. A scale change in PCs results in a rigid band shift of the Dirac-like cone, allowing for the manipulation of waves in pseudospin-1 systems in much the same way as applying a gate voltage in pseudospin-1/2 graphene. The transport of waves in pseudospin-1 systems exhibits many interesting phenomena, including super Klein tunneling, robust supercollimation, and unconventional Anderson localization. The transport properties of pseudospin-1 systems are distinct from their counterparts in pseudospin-1/2 systems, which will also be presented for comparison.

## 1. Introduction

Graphene has become a fertile platform to explore phenomena related to Dirac particles predicted in fundamental physics and to realize peculiar physical phenomena in materials science [1–15]. These interesting phenomena include unconventional half-integer quantum Hall effect in graphene when subjected to magnetic fields [4–6], Klein paradox that a classically forbidden region is transparent for Dirac electrons [7, 15], weak antilocalization due to the destructive interference between two counter-propagating backscattering waves [8, 9], Zitterbewegung of Dirac electrons in the presence of confining potentials [10, 11, 15], and supercollimation of electron wave packets in graphene subjected to one-dimensional (1D) disordered potentials [14]. The low-energy quasiparticles in graphene can be described by a massless Dirac equation and the wave function can be expressed in the form of a two-component spinor [15–17] due to the two sublattice degrees of freedom in graphene. As such, graphene is called a “pseudospin-1/2” system. We note that the spin here is not the intrinsic spin of electrons, but a pseudospin referring to the spatial degree of freedom of the wave function. Such pseudospin-1/2 systems can also be found in other systems [18–24], such as topological insulators in which the surface state dispersion exhibits a Dirac cone [18, 19], and photonic and phononic crystals in triangular or honeycomb lattices in which Dirac cones are found at the corners of the Brillouin zone [20–23].

With the rapid progress of experimental techniques, systems with higher pseudospin values, such as pseudospin-1, have been constructed using various artificial materials via a fine tuning of system parameters. A pseudospin-1 system is characterized by two linear bands meeting and intersecting with an additional flat band at a Dirac-like point. Such systems have attracted quite a bit of attention. The most typical lattice structure to realize pseudospin-1 systems is the Lieb lattice. Its sublattice symmetry protects the existence of a flat band. Pseudospin-1 systems have been realized experimentally in different artificially constructed systems. For example, it is found that the above Dirac-like dispersion can be realized at the corner of the Brillouin zone by placing bosonic cold atoms into an optical Lieb lattice [25–27]. In addition, a photonic Lieb lattice formed by an array of optical waveguides can also exhibit the Dirac-like dispersion [28–31]. More recently, two groups presented two different methods for realizing an electronic Lieb lattice through atom manipulation with a low-temperature scanning tunneling microscope (STM) [32, 33]. Drost et al. removed atoms from a chlorine layer placed on top of a Cu(100) crystal surface, leaving the desired Lieb lattice formed by the atomic vacancies [32]. In contrast, Slot et al. achieved the Lieb lattice not by vacancies but through adding carbon monoxide (CO) molecules to the top of a Cu(111) surface [33]. Theoretical works also predict that such a Dirac-like cone can be found in artificial crystals of ultracold atoms in Dice (T_3_) or stacked triangular lattices [34–40], and some electronic materials, such as transition-metal oxide SrTiO_3_/SrIrO_3_/SrTiO_3_ trilayer heterostructures [41], blue phosphorene oxide [42], and graphene-In_2_Te_2_ bilayer [43]. In those systems, the pseudospin-1 character emerges from the embedded sublattice degrees of freedom. In addition, some two-dimensional (2D) photonic crystals (PCs) are found to carry a Dirac-like cone at** k**=0 induced by the accidental degeneracy of monopole and dipole excitations [21, 22, 44–50], which combine to give three degrees of freedom. For such PCs, calculations using effective medium theory show that both effective permittivity and permeability reach zero at the Dirac-like point frequency [22, 44–50]. Above/below the Dirac-like point frequency, the effective parameters are positive/negative. Different pseudospin number leads to different boundary conditions and makes a marked difference on the transport properties of the pseudospin-1 systems. Many interesting transport phenomena, which are different from those in pseudospin-1/2 systems, have been predicted in pseudospin-1 systems. For example, in the presence of 1D potential barrier, there is a so-called super Klein tunneling effect for pseudospin-1 systems [26, 27, 35, 47, 48], that is unity transmission for all incident angles when the incident energy is half of the potential barrier. In a pseudospin-1 superlattice formed by a Kronig-Penney type of photonic potential, an electromagnetic (EM) wave packet can propagate in the superlattice without any distortion of shape [47]. We call such phenomenon “supercollimation”. When pseudospin-1 systems are subjected to 1D disordered potentials, two unconventional localization behaviors are found for obliquely incident waves. One is nonuniversal critical behavior for which the critical exponent of the localization length depends strongly on the type of disorder [51]. Another is the existence of a minimum localization length at some critical disorder strength beyond which the waves become less localized [52]. In the presence of a circular potential barrier, a perfect caustic phenomenon can occur for large scatterer size when the incident energy equals half of the barrier due to the super-Klein tunneling effect [53]. Furthermore, in the low-energy regime, a superscattering phenomenon occurs for an arbitrarily weak scatterer, i.e., extraordinarily strong scattering characterized by an unusually large cross section [54]. It was also reported that when a spatially uniform electric field is suddenly applied to an electronic pseudospin-1 system, the resulting current can be enhanced by the flat band in both the linear and nonlinear response regimes, compared with that in the pseudospin-1/2 system [55].

Unlike artificially ultracold atom and electronic Lieb lattices, PCs do not require extremely low temperature and atomic level manipulation. The photonic pseudospin-1 system was first experimentally demonstrated in the microwave frequency regime [44] and later in the optical regime [46]. The notion of pseudospin-1 was subsequently extended to aperiodic systems, as conical dispersions at** k**=0 can also be realized in some photonic quasicrystals as well as the effective zero refractive index [50].

Since pseudospin-1 systems were proposed to realize using artificially constructed ultracold-atom systems around nine years ago, many new interesting phenomena have been discovered in the past few years as mentioned before. While recent experiments demonstrate the viability of realizing pseudospin-1 systems with ultracold-atom or electronic Lieb lattices, it still remains challenging to achieve a long enough coherence length, which makes it difficult to have complex junctions in such systems to observe the theoretically predicted novel behaviors experimentally. Thus, in the review, we give a comprehensive review of the theoretical predictions and experimental progress on the pseudospin-1 systems with a focus on the newly discovered phenomena in PC-based systems. Due to the ease of fabrication and absence of complex interactions between photons, these phenomena may finally be tested in systems formed by PCs. In Section 2, we introduce the relation between the pseudospin-1 dielectric PCs and the zero refractive index effective media, and the experimental realizations of such PCs. In Section 3, we show the calculation of the Berry phase for a loop encircling the Dirac-like point. In Section 4, we relate the Dirac-like cone dispersion of PCs to the spin-orbit Hamiltonian description of pseudospin-1 based on the zero refractive index effective medium description, and we show the photonic counterpart of the gate voltage in graphene. The 1D transport phenomena in pseudospin-1 systems are reviewed in Section 5, including Klein tunneling, supercollimation and unconventional Anderson localization behaviors in disordered 1D potentials for oblique incident angles. A brief summary is given in Section 6.

## 2. Effective Medium Description of Pseudospin-1 PCs

**Figure 1:**
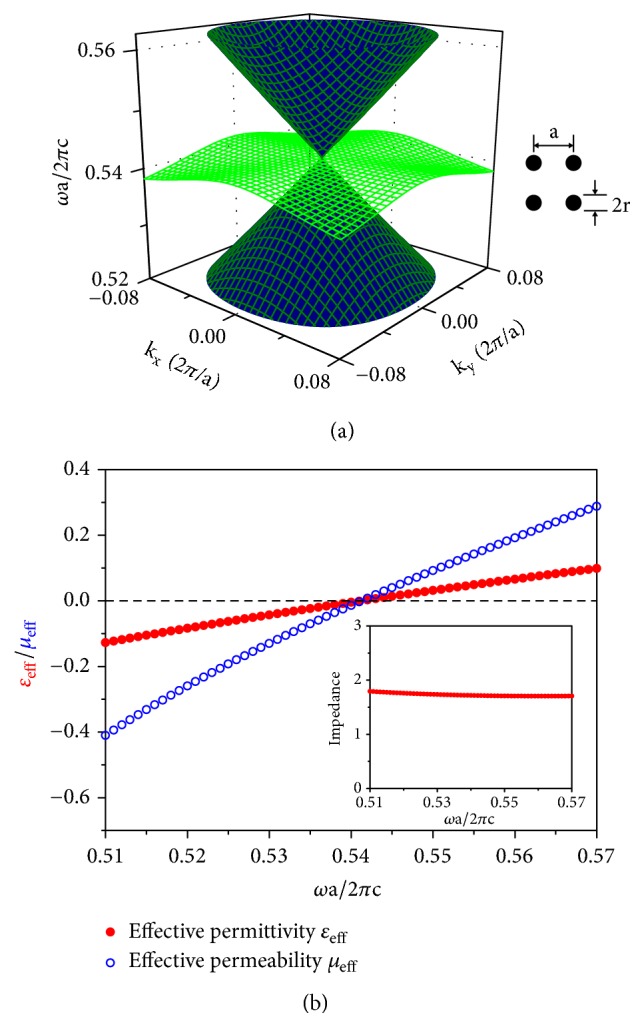
(a) The Dirac-like cone dispersion near the Γ point (**k**=0) calculated for a 2D square lattice PC with dielectric cylinders in air. The permittivity and radii of the cylinders are *ε* = 12.5 and *r* = 0.2*a* (*a* is the lattice constant), and c is the speed of light in vacuum. The inset shows the unit cell of the PC. Adapted from [47]. (b) The frequency dependence of the effective permittivity (*ε*_*eff*_) and permeability (*μ*_*eff*_) near the Dirac-like point frequency *ω*_*D*_. The inset shows the nearly constant effective impedance obtained from *ε*_*eff*_ and *μ*_*eff*_.

It is well known that the lattice symmetry of the 2D PCs with a triangular lattice guarantees the existence of Dirac cones at the Brillouin zone corners with a pseudospin-1/2 character [20–23]. However, effective medium theory is only good for small** k** vectors (long wavelength) and is not applicable in general for Dirac cones located at the Brillouin zone boundary. At the Brillouin zone center, the lattice symmetry alone gives quadratic dispersions only. It is found that for some 2D dielectric PCs with C_4v_ or C_3v_ symmetries, e.g., square or triangular lattices, the monopole and dipole excitations can become triply degenerate by fine tuning of the radii or permittivity of cylinders so that a Dirac-like cone appears at** k**=0, in which the conical dispersions intersect with an additional flat band at the Dirac-like point [22, 44, 45]. Such degeneracy is not given by the lattice symmetry. Instead, it occurs only for some specific values of the radius and permittivity of the dielectric rods composed of the PCs and hence the degeneracy is accidental. As an example, in Figure 1(a), we show the three eigenfrequency surfaces near** k**=0 calculated for the transverse electric (TE) modes for a 2D square lattice PC with dielectric cylinders in air. Here, TE modes refer to the EM modes with the electric field along the axis of cylinders. The inset of Figure 1(a) shows the unit cell of the PC. The permittivity and radii of the dielectric cylinders are taken as *ε* = 12.5 and *r* = 0.2*a* (*a* is the lattice constant). It can be clearly seen that near** k**=0, a Dirac cone (blue) meets and intersects with a nearly flat band (green) at the Dirac-like point frequency. The modes in the Dirac cone are transverse EM modes with a finite group velocity, while those modes in the flat band are longitudinal EM modes with the locally averaged magnetic field vector aligned with the** k**-vector and having nearly zero group velocity [44, 45].

The optical properties of such 2D PCs exhibiting a Dirac-like cone near** k**=0 is the same as an effective medium with zero refractive index at the Dirac-like point frequency. In Figure 1(b), we show the effective permittivity (*ε*_*eff*_) and permeability (*μ*_*eff*_) near the Dirac-like point frequency of the PC calculated by the effective medium theory [56]. We note that both *ε*_*eff*_ (red solid circles) and *μ*_*eff*_ (blue open circles) reach zero at the Dirac-like point frequency *ω*_*D*_ = 1.0826*π*(*c*/*a*) (c is the speed of light in vacuum), and their dispersions are linear with a frequency change given by *δω* = *ω* − *ω*_*D*_, giving rise to a nearly constant effective impedance as shown in the inset of Figure 1(b). As we will show later, these features are very important when we map the Dirac-like cone dispersion at** k**=0 onto a spin-orbit Hamiltonian of pseudospin 1 based on the effective medium description. It should be pointed out that the effective material constitutive parameters of 2D PCs are anisotropic. If we assume that the PCs are arranged periodically in the* xy* plane and the rods are along* z*-direction, the effective permittivity and permeability can be described by diagonal tensors $\overset{⃡}{\varepsilon }=diag(\varepsilon_{xx},\varepsilon_{yy},\varepsilon_{zz})$ and $\overset{⃡}{\mu }=diag(\mu_{xx},\mu_{yy},\mu_{zz})$, respectively, with *ε*_*xx*_ = *ε*_*yy*_ and *μ*_*xx*_ = *μ*_*yy*_ for triangular and square lattices [57]. For TE modes, only *ε*_*zz*_, *μ*_*xx*_, and *μ*_*yy*_ enter the wave propagation problem. Thus, the effective parameters *ε*_*eff*_ and *μ*_*eff*_ shown in Figure 1(b) are actually *ε*_*eff*_ = *ε*_*zz*_ and *μ*_*eff*_ = *μ*_*xx*_ = *μ*_*yy*_. Compared with a single zero material (*ε*_*eff*_ = 0 or *μ*_*eff*_ = 0 but not both), the double-zero material here has a finite impedance, which is a desirable feature in eliminating strong reflections from the interface.

The PC with *ε*_*eff*_ = *μ*_*eff*_ = 0 was first realized experimentally in the microwave regime by arranging alumina rods (*r* = 3.75mm and *ε* = 8.8) in a square lattice (*a* = 17mm) in air [44]. Using this PC structure, some unique properties arising from zero refractive index were demonstrated experimentally, such as cloaking and lensing [44]. The PC at optical frequencies was implemented by a stack of square-cross-section silicon rods (*ε* = 13.7, width=260 nm, and *a* = 600nm) embedded in SiO_2_ (*ε* = 2.25) [46]. The experimentally observed angular selectivity of transmission and directive spontaneous emission from quantum dots placed within the material provide direct evidence that the PC has indeed a near-zero refractive index near the Dirac-like point frequency [46]. We note here that since the PC is all dielectric, it is much less absorptive than conventional metal-based metamaterials in which the metallic components induce large ohmic loss at high frequencies. Periodicity is not a necessary condition to realize the Dirac-like cone at** k**=0. The Dirac-like cone dispersion and zero refractive index were also realized experimentally in a 2D 12-fold photonic quasicrystal constructed by placing dielectric rods at the 2D dodecagonal lattice sites generated by the square-triangle tiling model [50].

## 3. Berry Phase of the Dirac-Like Cone

It is well known that the Dirac cone of graphene and its photonic counterpart give rise to a Berry phase of *π* [15, 58]. It is interesting to know if the additional flat band affects the Berry phase of the Dirac-like cone. In general, one can calculate numerically the eigenmodes of the PC to obtain the Berry phase. For square or triangular lattice PCs composed of cylindrical rods, one can conveniently use the multiple scattering theory (MST) to obtain the eigenmodes [44, 45]. For the Dirac-like cones studied in this review, it is sufficient to consider the monopolar (*m* = 0) and dipolar (*m* = ±1) terms only [44]. Here* m* denotes the angular momentum number of the eigenmode. The MST equations can be expressed as [44]

(1) $$\begin{array}{c} \left(\begin{array}{ccc} S_{0}-\frac{1}{D_{-1}} & -S_{1} & S_{2}\\ -S_{-1} & S_{0}-\frac{1}{D_{0}} & -S_{1}\\ S_{-2} & -S_{-1} & S_{0}-\frac{1}{D_{1}} \end{array}\right)\left(\begin{array}{c} b_{-1}\\ b_{0}\\ b_{1} \end{array}\right)=0, \end{array}$$

where *D*_*m*_ and *b*_*m*_ are the* T*-matrix coefficients and the mode amplitudes of Mie scattering, respectively, and *S*_*m*_ denotes the lattice sum with *S*_−*m*_ = −*S*_*m*_^*∗*^, with *m* = 0, ±1. At the Dirac-like point, where *ω* = *ω*_*D*_ and** k** = 0, we have *S*_0_ = 1/*D*_0_ = 1/*D*_±1_ [44]. Near the Dirac-like point, one can take Taylor expansions with respect to** k** and *ω* for *S*_*m*_ and *D*_*m*_ (*m* = 0, ±1) up to the first order of** k** and *δω* = *ω* − *ω*_*D*_. Thus, one can obtain *S*_0_ − 1/*D*_0_ ≈ *iA*_0_ · (*ω* − *ω*_*D*_), *S*_0_ − 1/*D*_±1_ ≈ *iA*_1_ · (*ω* − *ω*_*D*_), *S*_1_ ≈ *C*_1_*ke*^*iϕ*_**k**_^ and *S*_±2_ ≈ 0, where *A*_0_, *A*_1_, and *C*_1_ are real, and *k* and *ϕ*_**k**_ are the magnitude and angle of** k** in the polar coordinate [44, 45]. By letting the determinant of the 3 × 3 matrix in (1) equal to zero, one can obtain three solutions: *ω* − *ω*_*D*_ = *sv*_*g*_*k* with *s* = 0, ±1 and $v_{g}=\sqrt{2}|C_{1}|/\sqrt{A_{0}A_{1}}$. When *s* = 0, the eigenvector is $\left|\Phi_{s,k}\rangle \right.={\left(\begin{array}{ccc} e^{i\phi_{k}} & 0 & e^{-i\phi_{k}} \end{array}\right)}^{T}$, which corresponds to the flat band. When *s* = ±1, the eigenvectors are $\left|\Phi_{s,k}\rangle \right.={\left(\begin{array}{ccc} -isC_{1}e^{i\phi_{k}}/A_{1}v_{g} & 1 & isC_{1}e^{-i\phi_{k}}/A_{1}v_{g} \end{array}\right)}^{T}$, corresponding to the two linear bands [45]. Using the three eigenvectors, one can obtain the Berry phase for each band of the Dirac-like cone; that is, *γ* = ∮*i*〈Φ_*s*,**k**_∣∇_**k**_Φ_*s*,**k**_〉 · *d ***k** = 0 [45], which is different from that of the Dirac cone in grapheme [15].

## 4. Pseudospin-1 Description of a Dirac-Like Cone in PCs

### 4.1. Spin-Orbit Hamiltonian Near **k** = 0 from Maxwell's Equations

We start from the Maxwell's equations and apply effective medium theory to demonstrate that the Dirac-like conical dispersion of PCs near** k** = 0 can be related to an effective spin-orbit Hamiltonian of pseudospin 1. We will assume that bands forming the Dirac cone are derived from monopole and dipole excitations and under those circumstances, we can safely apply effective medium theory to describe EM wave propagation in 2D PCs near a Dirac-like point at the Brillouin zone center. The waves are assumed to be TE modes propagating in the effective medium (*xy* plane) of 2D PCs shown in Section 2, with electric field along* z*-direction and magnetic field in the* xy* plane. As we discussed in Section 2, for the effective material constitutive tensors of PCs, we only need to take into consideration *ε*_*zz*_, *μ*_*xx*_, and *μ*_*yy*_. We note that all three parameters are functions of frequency *ω*. By letting *ε*_*zz*_ = *ε* and *μ*_*xx*_ = *μ*_*yy*_ = *μ*, the Maxwell's equations can be expressed in the following matrix form [47]:

(2) 0−i∂∂x−∂∂y0−i∂∂x+∂∂y0−i∂∂x−∂∂y0−i∂∂x+∂∂y0ψ~=ωμ0002ε000μψ~,

where $\tilde{\psi }$ is ${\tilde{\psi }}_{T}=(-iH_{x}-H_{y},E_{z},iH_{x}-H_{y}{)}^{T}$ for transverse modes and ${\tilde{\psi }}_{L}=(H_{x}-iH_{y},E_{z},-H_{x}-iH_{y}{)}^{T}$ with *E*_*z*_ being spatially independent for longitudinal modes, respectively. We note that $\tilde{\psi }$ are different from the eigenvectors of PCs in Section 3, where the three components correspond to the mode amplitudes of Mie scattering with the angular momentum number* m*=-1, 0, and 1, respectively. As shown in Section 2, for PCs with a conical dispersion at** k** = 0, the effective permittivity *ε* and permeability *μ* approach linearly to zero at the Dirac-like point frequency *ω*_*D*_, and we have $\omega \varepsilon ≅(\omega -\omega_{D})\tilde{\varepsilon }$ and $\omega \mu ≅(\omega -\omega_{D})\tilde{\mu }$ in the neighborhood of *ω*_*D*_ [44, 47]. The system parameters $\tilde{\varepsilon }{\left.=\omega_{D}(d\varepsilon /d\omega )\right|}_{\omega =\omega_{D}}$ and $\tilde{\mu }{\left.=\omega_{D}(d\mu /d\omega )\right|}_{\omega =\omega_{D}}$ must be positive definite in order for the energy density to be nonnegative [44, 47].

Through the Fourier transform of $\tilde{\psi }$ in* k*-space, i.e., ${\tilde{\psi }}^{\left(k\right)}=(1/2\pi )\int \tilde{\psi }e^{-ik\cdot r}dr$, we get ${\tilde{\psi }}_{T}^{\left(k\right)}=(-iH_{x}^{\left(k\right)}-H_{y}^{\left(k\right)},E_{z}^{\left(k\right)},iH_{x}^{\left(k\right)}-H_{y}^{\left(k\right)}{)}^{T}$ for transverse modes and ${\tilde{\psi }}_{L}^{\left(k\right)}=(H_{x}^{\left(k\right)}-iH_{y}^{\left(k\right)},E_{z}^{\left(k\right)},-H_{x}^{\left(k\right)}-iH_{y}^{\left(k\right)}{)}^{T}$ for longitudinal modes, with *H*_*m*_^(**k**)^ = (1/2*π*)∫*H*_*m*_*e*^−*i ***k**·**r**^*d ***r** (*m* = *x*, *y*), and *E*_*z*_^(**k**)^ = (1/2*π*)∫*E*_*z*_*e*^−*i ***k**·**r**^*d ***r**. Using the above linear approximations of *ωε* and *ωμ*, (2) can be transformed to the following equation in* k*-space

(3) 0kx−iky0kx+iky0kx−iky0kx+iky0ψ~k=δωμ~0002ε~000μ~ψ~k,

where *δω* = *ω* − *ω*_*D*_, and *k*_*x*_ (*k*_*y*_) is the projection of the 2D wavevector** k** on the* x* (*y*) axis. We note that, for longitudinal modes with any nonzero** k**, the component *E*_*z*_^(**k**)^is zero. Using a similarity transformation with $U=diag\left(2\sqrt{\tilde{\varepsilon }/\tilde{\mu }},\sqrt{2},2\sqrt{\tilde{\varepsilon }/\tilde{\mu }}\right)$ and ${\tilde{\psi }}^{\left(k\right)}=U\psi^{\left(k\right)}$, we can rewrite (3) as

(4) 12ε~μ~0kx−iky0kx+iky0kx−iky0kx+iky0ψk=δωψk.

As can be seen in Figure 1(b), the effective constitutive parameters of the PCs are negative (*ε*, *μ* < 0) below the Dirac-like point frequency *ω*_*D*_, while they are positive (*ε*, *μ* > 0) above *ω*_*D*_. Right at *ω*_*D*_, the effective refractive index is zero (*ε* = *μ* = 0). The sign change of *δω* in (4) is directly related to the sign change of *ε* and *μ* in (2) when the frequency crosses over *ω*_*D*_. Thus, the positive and negative index behaviors of the system are treated by (4) in a unified framework. Equation (4) can be further rewritten as *Hψ*^(**k**)^ = *δωψ*^(**k**)^ with

(5) $$\begin{array}{c} H=v_{g}S\cdot k, \end{array}$$

by using the following spin-1 matrices, i.e., $S=S_{x}\hat{x}+S_{y}\hat{y}$ with

(6) Sx=12010101010,Sy=120−i0i0−i0i0.

Here $v_{g}=1/\sqrt{\tilde{\varepsilon }\tilde{\mu }}$ is the group velocity of the conical dispersion [47]. Equation (4) or (5) represents a spin-orbit interaction of pseudospin 1. Three normalized eigenvectors can be obtained for (4) or (5):

(7) ψ−sk=12se−iθk2seiθks=±1and  ψ−sk=12e−iθk0−eiθks=0.

Here *θ*_**k**_ is the angle between the wavevector** k **and the positive* x*-direction. The three bands of the Dirac-like cone can be clearly seen from the corresponding eigenvalues *δω* = *sv*_*g*_|**k**| (*s* = 0, ±1). Here *s* = ±1 correspond to the upper (*s* = +1) and lower (*s* = −1) conical bands, while *s* = 0 corresponds to the flat band. The *s* values also represent three pseudospin states in which the pseudospin is either parallel (*s* = +1), perpendicular (*s* = 0), or antiparallel (*s* = −1) to the wavevector** k**. The chiralities of the upper and lower cones are opposite. From (7), we can obtain that the Berry phase *γ* equals 0 for a loop around the Dirac-like cone, according to the formula $\gamma =i\oint \langle \left.{\overset{-}{\psi }}_{s}^{\left(k\right)}\right|\nabla_{k}\left|{\overset{-}{\psi }}_{s}^{\left(k\right)}\rangle \right.\cdot dk$. This is consistent with the results obtained from the MST theory for a realistic PC structure shown in Section 3. For comparison, the corresponding Berry phase is *π* for pseudospin-1/2 systems (e.g., graphene). This shows immediately that while both pseudospin-1 and pseudospin-1/2 systems are characterized by conical dispersions, the physics involving wave scattering are quite different. Next, we will show that the normalized eigenvector ${\overset{-}{\psi }}_{+1}^{\left(k\right)}$ in (7) corresponds to a transverse mode in a positive medium (*ε*, *μ* > 0), while ${\overset{-}{\psi }}_{-1}^{\left(k\right)}$ corresponds to one in a negative medium (*ε*, *μ* < 0). For the TE modes under investigations, the electric field *E*_*z*_^(**k**)^ in the normalized eigenvectors ${\overset{-}{\psi }}_{s}^{\left(k\right)}\;\;$(*s* = ±1) is taken as 1. For arbitrary values of *E*_*z*_^(**k**)^, we have $\psi_{T}^{\left(k\right)}=E_{z}^{\left(k\right)}{\overset{-}{\psi }}_{\pm 1}^{\left(k\right)}$. From the Maxwell's equations, the magnetic field **H**^(**k**)^ can be expressed as $H^{\left(k\right)}=H_{x}^{\left(k\right)}\hat{x}+H_{y}^{\left(k\right)}\hat{y}$ with *H*_*x*_^(**k**)^ = (*k*_*y*_/*ωμ*)*E*_*z*_^(**k**)^ and *H*_*y*_^(**k**)^ = −(*k*_*x*_/*ωμ*)*E*_*z*_^(**k**)^. Using the first-order approximation of *ωμ*, i.e., $\omega \mu \approx (\omega -\omega_{D})\tilde{\mu }$ and the linear dispersion *ω* − *ω*_*D*_ = *sv*_*g*_ | **k**|, we can write $H_{x}^{\left(k\right)}\approx s(sin\theta_{k}/v_{g}\tilde{\mu })E_{z}^{\left(k\right)}$ and $H_{y}^{\left(k\right)}\approx -s(cos\theta_{k}/v_{g}\tilde{\mu })E_{z}^{\left(k\right)}$, i.e., $H^{\left(k\right)}=s\hat{k}\times E^{\left(k\right)}/(v_{g}\tilde{\mu })$ for *s* = ±1, where $\hat{k}=k/\left|k\right|$ is a unit vector indicating the direction of wavevector** k**. We see that *s* = +1 describes a transverse mode in positive media for which the three vectors **E**^(**k**)^, **H**^(**k**)^, and $\hat{k}$ form a right-handed triad, while *s* = −1 describes one in negative media for which they form a left-handed triad. In terms of electromagnetic fields, the eigenvectors are rewritten as

(8) $$\begin{array}{c} \psi_{T}^{\left(k\right)}=E_{z}^{\left(k\right)}{\overset{-}{\psi }}_{\pm 1}^{\left(k\right)}=\frac{1}{2}\left(\begin{array}{c} -\sqrt{\tilde{\mu }/\tilde{\varepsilon }}\left(iH_{x}^{\left(k\right)}+H_{y}^{\left(k\right)}\right)\\ \sqrt{2}E_{z}^{\left(k\right)}\\ \sqrt{\tilde{\mu }/\tilde{\varepsilon }}\left(iH_{x}^{\left(k\right)}-H_{y}^{\left(k\right)}\right) \end{array}\right). \end{array}$$

When *s* = 0, the eigenvector ${\overset{-}{\psi }}_{s}^{\left(k\right)}$corresponds to a longitudinal mode. In terms of electromagnetic fields, it can be expressed as [47],

(9) $$\begin{array}{c} \psi_{L}^{\left(k\right)}=\left(\begin{array}{c} H_{x}^{\left(k\right)}-iH_{y}^{\left(k\right)}\\ 0\\ -H_{x}^{\left(k\right)}-iH_{y}^{\left(k\right)} \end{array}\right). \end{array}$$

It should be mentioned that in our effective medium description the group velocity of the flat band is zero. However, in realistic PC structures, for** k** near the Dirac-like point, the group velocity can be small and negligible. When** k** is far away from the Dirac-like point, the effective medium description fails so that the band is not flat any more, i.e., it has finite group velocity and the band modes are not longitudinal modes either. Due to the orthogonality between the transverse and longitudinal modes, these flat band modes near the Dirac-like point are almost impossible to be excited by incidence of a propagating transverse mode in experiments. Thus, we ignore these longitudinal modes when we study the transport properties of the transverse waves in Section 5. To detect these flat band modes experimentally, one may put a point source inside the 2D PCs. In this case, a localized excitation appears in the vicinity of the source, which moves with the position of source. For ultracold atoms in a Lieb lattice, the atoms can be transferred coherently into the flat band by actively engineering the population and phase on each lattice site [25]. For an array of optical waveguides with a Lieb lattice configuration, the flat band mode can be excited by a structured excitation of the three sublattices [28–31].

### 4.2. Photonic Counterpart of Gate Voltage

It is well known that electron transport in graphene can be manipulated by applying a gate voltage to form complex junctions, with which many interesting transport phenomena have been observed experimentally or predicted theoretically, such as Klein tunneling [7, 15] and supercollimation of electron wave packets [12–14]. One might be interested to know whether we can find the photonic counterpart of gate voltage in graphene which can shift the Dirac-like cones rigidly up or down in frequency with the group velocity essentially unchanged, and whether the application of such photonic potentials in PCs will result in any new physical phenomena. It is also well known in classical wave physics that one possible way to manipulate the propagation of waves is to make the structural variation in the system to form a certain structural pattern. We will see that, for dielectric PCs, a particular kind of structural change is equivalent to applying a gate voltage in electrons. Such structurally induced photonic potential will play a significant role in the dynamics of pseudospin-1 wave packets.

Here we show that such a frequency upshift or downshift of the Dirac-like cone can be easily achieved by changing the length scale of a dielectric PC [47]. Suppose that a dielectric structure with a spatial configuration of dielectric *ε*(**r**) is scaled uniformly by a factor *s* to achieve a new configuration *ε*′(**r**) = *ε*(**r**/*s*), the mode frequency *ω*′ and wavevector **k**′ of the new configuration can be obtained by rescaling the mode frequency *ω* and wavevector **k** of the old one through the relations *ω*′ = *ω*/*s* and **k**′ = **k**/*s*, according to the scaling properties of the Maxwell's equations [59]. We assume that the materials consisting of the structure have frequency-independent permittivity in the operational frequency range. It can be seen clearly from the scaling relations that the change of length scale can shift up/down the Dirac-like point frequency, Δ*ω*_*D*_ = *ω*′_*D*_ − *ω*_*D*_ = [1/*s* − 1]*ω*_*D*_. Here *ω*_**D**_ and *ω*′_**D**_ indicate the respective Dirac-like point frequencies of the original and scaled PCs. More importantly, the scaling does not change the group velocity of the conical dispersion,

(10) $$\begin{array}{c} {v^{\prime }}_{g}=\nabla_{k^{\prime }}\omega^{\prime }=\nabla_{sk^{\prime }}\left(\;s\omega^{\prime }\;\right)=\nabla_{k}\omega =v_{g}, \end{array}$$

where **v**_**g**_ and **v**′_**g**_ indicate the respective group velocities before and after the scaling. The change of length scales of dielectric PCs plays the role of the gate voltage in graphene, shifting rigidly the conical dispersions of PCs in frequency. As a photonic analog of electron potential, the length-scaling induced Dirac-like point frequency shift (Δ*ω*_**D**_) is effectively a photonic gate potential *V* with *V* = Δ*ω*_*D*_ = *ω*′_*D*_ − *ω*_*D*_. If the local length scales of the PCs are modulated along one direction, for example, the* x*-direction, we can construct a 1D photonic potential *V*(*x*) = *ω*′_*D*_(*x*) − *ω*_*D*_. The total Hamiltonian now becomes

(11) $$\begin{array}{c} H=v_{g}S\cdot k+V\left(\;x\;\right)I. \end{array}$$

**I** is a 3 × 3 identity matrix. From (11), pseudospin-1 waves propagating along the* x*-direction do not experience backscattering from the 1D photonic potential, which leads to one-way transport phenomenon. From the EM wave point of view, this is a result of impedance matching because the impedance is a scale invariant constant near the Dirac-like point frequency, as shown analytically in [47] and numerically in the inset of Figure 1(b).

To see how pseudospin-1 waves are scattered by 1D potential, we need to consider the boundary conditions at the interface. Assuming that *ψ* = (*ψ*_1_, *ψ*_2_, *ψ*_3_)^*T*^is a general solution of (11), we can obtain three boundary conditions at an interface *x* = *x*_0_ by integrating the wave equation *Hψ* = *δωψ* over a small interval *x* ∈ [*x*_0_ − *η*, *x*_0_ + *η*] along the* x*-direction and taking the limit *η* → 0 [27, 35, 47],

(12) ψ2x0−η=ψ2x0+η,

(13) ψ1x0−η+ψ3x0−η=ψ1x0+η+ψ3x0+η,

(14) δω−Vx0−ηψ3x0−η−ψ1x0−η=δω−Vx0+ηψ3x0+η−ψ1x0+η.

If the wave function takes the form of (8) or (9), we directly obtain that (12)-(14) are equivalent to the continuity of *E*_*z*_, *H*_*y*_, and *μH*_*x*_, respectively, i.e., the boundary conditions required by TE waves. We note here that only the first two boundary conditions, i.e., Eqs. (12) and (13), are independent because the continuity of *ψ*_2_ implies the continuity of [*δω* − *V*][*ψ*_3_ − *ψ*_1_] [47]. It is consistent with EM wave theory in which, for time-harmonic fields, the continuity of the tangential** E **and** H** components across interfaces implies the continuity of normal** B** and** D** components [60].

## 5. Transport Properties of Pseudospin-1 Waves in 1D Potentials

### 5.1. Klein Tunneling of Pseudospin-1 EM Waves

**Figure 2:**
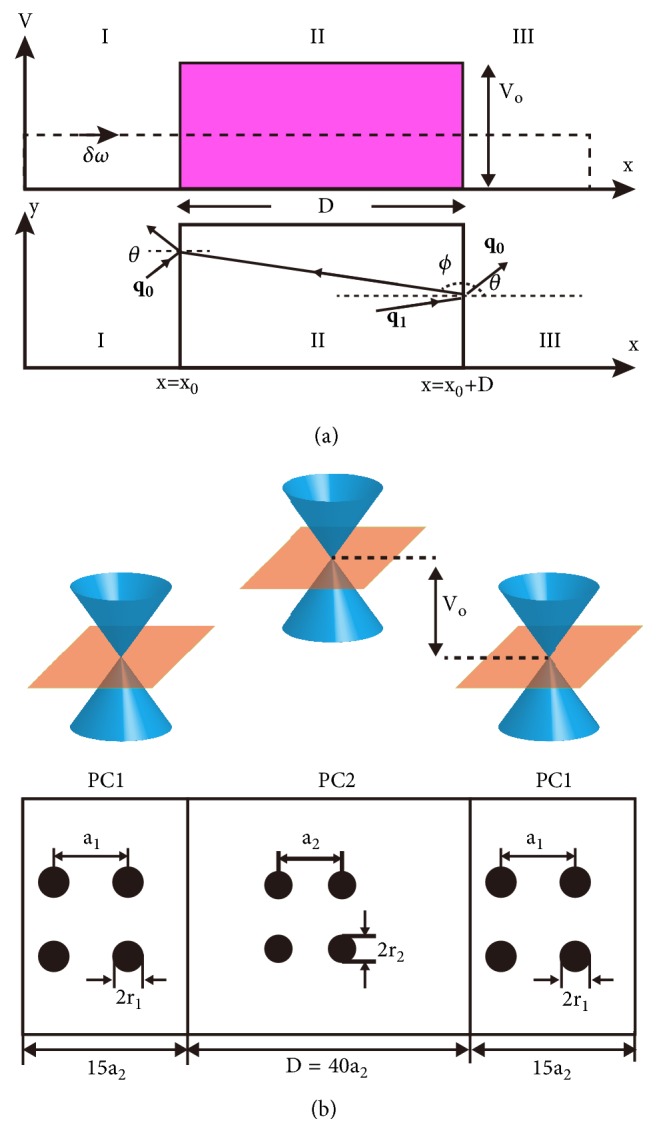
(a) Schematic diagram illustrating the scattering of an incident wave by a square photonic potential barrier. (b) A square photonic potential barrier constructed by a PC sandwich structure. Both PC1 and PC2 are square lattice dielectric photonic crystals, but with different length scales (*a*_1_ = 15*a*_2_/14 and *r*_1_ = 15*r*_2_/14). The PC1 and PC2 domains have a thickness of 15*a*_2_ and 40*a*_2_, respectively. The length scale change induces a rigid band shift of the conical dispersion. Adapted from [47].

We now consider the scattering of pseudospin-1 EM waves travelling in the* xy* plane by a 1D square photonic potential barrier. The scattering process is schematically depicted in Figure 2(a). The wave functions in the three regions of Figure 2(a) can be written in terms of the eigenvectors in (7). The wave function in region I can be expressed as

(15) ψI=a~02se−iθ2seiθeiq0xx+q0yy+b~02se−iπ−θ2seiπ−θei−q0xx+q0yy,

with *s* = sgn(*δω*), *θ* = arctan(*q*_0*y*_/*q*_0*x*_), *q*_0*x*_ = |**q**_0_|cos⁡*θ*, *q*_0*y*_ = |**q**_0_|sin⁡*θ*, and |**q**_0_ | = |*δω* | /*v*_*g*_. In region II, we have

(16) ψII=c~2s′e−iϕ2s′eiϕeiq1xx+q0yy+d~2s′e−iπ−ϕ2s′eiπ−ϕei−q1xx+q0yy,

with *ϕ* = *π* + arctan(*q*_0*y*_/*q*_1*x*_), *s*′ = sgn(*δω* − *V*_0_), and $q_{1x}=-\sqrt{(V_{0}-\delta \omega^{2}{)}^{2}/(v_{g}^{2})-q_{0y}^{2}}$. In region III, we have

(17) ψIII=a~12se−iθ2seiθeiq0xx+q0yy+b~12se−iπ−θ2seiπ−θei−q0xx+q0yy.

A transfer matrix **M** can be defined by the relation,

(18) $$\begin{array}{c} \left(\begin{array}{c} a_{1}\\ b_{1} \end{array}\right)=M\left(\begin{array}{c} a_{0}\\ b_{0} \end{array}\right), \end{array}$$

where $a_{0}={\tilde{a}}_{0}e^{iq_{0x}x_{0}}$, $b_{0}={\tilde{b}}_{0}e^{-iq_{0x}x_{0}}$, $a_{1}={\tilde{a}}_{1}e^{iq_{0x}(x_{0}+D)}$and $b_{1}={\tilde{b}}_{1}e^{-iq_{0x}(x_{0}+D)}$. Here *x* = *x*_0_ and *x* = *x*_0_ + *D* are the left and right boundaries of the square barrier, respectively [see Figure 2(a)]. The transfer matrix can be obtained from the boundary conditions shown in (12) and (13) as

(19) $$\begin{array}{c} M\left(\;D\;\right)=\left(\begin{array}{cc} \alpha \left(D\right) & \beta \left(-D\right)\\ \beta \left(D\right) & \alpha \left(-D\right) \end{array}\right), \end{array}$$

with the elements,

(20) αD=cos⁡q1xD+i2ss′sin⁡q1xDcos⁡θcos⁡ϕ+cos⁡ϕcos⁡θ,βD=i2ss′sin⁡q1xDcos⁡θcos⁡ϕ−cos⁡ϕcos⁡θ.

We can obtain the transmission through the barrier from the transfer matrix** M**,

(21) Tθ=cos2ϕ cos2θcos⁡q1xDcos⁡ϕcos⁡θ2+sin2q1xD/4cos2ϕ+cos2θ2.

For normal incidence (*θ* = 0), it is obvious that *T*(*θ* = 0) = 1 for any value of *δω*. Such perfect transmission phenomenon at normal incidence is a manifestation of Klein tunneling effect in pseudospin-1 systems. For pseudospin-1 EM waves, the perfect transmission can be interpreted as a consequence of impedance matching at normal incidence. At a finite incident angle, for values of *q*_1*x*_*D* satisfying the relation *q*_1*x*_*D* = *mπ*  (*m* ∈ integers), we will have *T*(*θ*) = 1 due to Fabry-Perot resonances. More interestingly, when *δω* = *V*_0_/2, we have *q*_1*x*_ = −*q*_0*x*_ and *ϕ* = *π* − *θ*, and then obtain *T*(*θ*) = 1 for any incident angle. Such all-angle transparency is called “super Klein tunneling”, which can only happen for pseudospin-1 systems.

The super Klein tunneling effect was verified numerically using a realistic PC sandwich structure [47]. It can also be understood from the standpoint of impedance matching in EM wave theory. When *δω* = *V*_0_/2, the permittivities and permeabilities in the background and potential barrier are equal in magnitude but opposite in sign [47], thus the impedance is matched for all incident angles, which in turn leads to all-angle unity transmission. In EM wave theory, it is known that two adjacent layers, in case that they have equal thickness and their permittivities and permeabilities are equal in magnitude but opposite in sign, can “optically cancel” each other in space, and are called “complementary materials” in the metamaterial literature [61]. Interestingly, one may take the pseudospin-1 photonic system as a candidate to realize “complementary materials”. We note that “complementary materials” were extensively studied in the field of metamaterials which typically require the use of metallic inclusions to achieve the effective negative medium in order to cancel the optical path in a positive medium. However, the huge ohmic loss of metals at high frequencies makes it not so promising to realize them at optical frequencies. Pseudospin-1 photonic system is hence a good candidate since the PCs are all dielectric so that they have low loss even at high frequencies. Recently, experimental realization of complementary materials has been demonstrated in the microwave regime using PCs composed of alumina (*ε* = 8.1) rods in air [62, 63]. It looks promising to push the working frequency to the optical regime in the near future.

**Figure 3:**
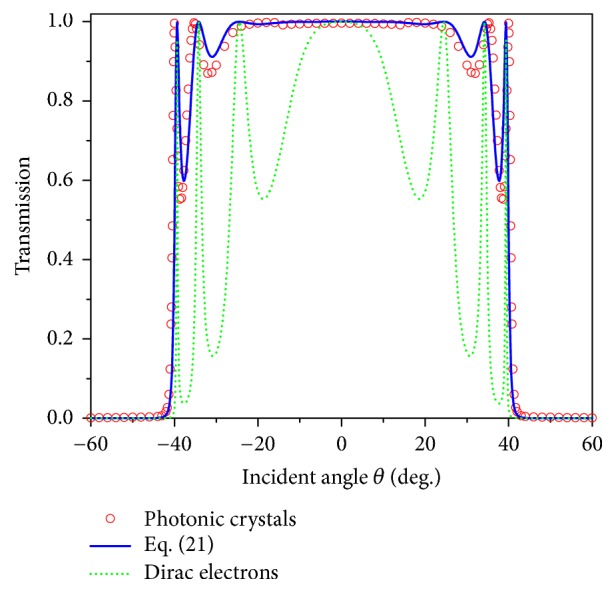
Transmissions calculated for a PC sandwich structure (red open circles), using (21) (blue solid line) and for Dirac electrons in graphene (green dotted line). The photonic potential *V*_0_ and group velocity *v*_*g*_ in (21) are obtained from the PC sandwich structure shown in Figure 2(b), and the reduced frequency *δω* is taken to be *δω* = 0.0436*πc*/*a*_2_. For Dirac electrons, the electron energy *E* satisfies *E*/*ħv*_*F*_ = *δω*/*v*_*g*_ while potential height *U*_0_ meets *U*_0_/*ħv*_*F*_ = *V*_0_/*v*_*g*_, with *v*_*F*_ being the Fermi velocity of Dirac electrons. The barrier width is *D* = 40*a*_2_.

We will show below how pseudospin-1 EM waves are scattered when they meet a 1D potential barrier in the case that *δω* deviates from *V*_0_/2. To study the scattering properties in realistic PC systems, a photonic potential barrier can be constructed using a PC sandwich structure. As shown in Figure 2(b), the sandwich structure is composed of two types of PCs, labelled as PC1 and PC2. Both of them are 2D square lattices of dielectric cylinders embedded in air. The dielectric constant *ε* of the cylinders is 12.5. The radii of the cylinders in the two PCs are *r*_*i*_ = 0.2*a*_*i*_  (*i* = 1,2) with *a*_1_ = (15/14)*a*_2_, where *a*_1_ and *a*_2_ are the lattice constants of PC1 and PC2, respectively. With the choice of the above parameters, both PCs exhibit a Dirac-like cone near** k** = 0 with the group velocity *v*_*g*_ = 0.2962*c*, where *c* is the speed of light in vacuum. The Dirac-like point frequencies of PC1 and PC2 are *ω*_*D*1_ = 1.0826*π*(*c*/*a*_1_) and *ω*_*D*2_ = 1.0826*π*(*c*/*a*_2_), respectively. The effective photonic potential shift between PC1 and PC2 is *V*_0_ = *ω*_*D*2_ − *ω*_*D*1_ = (1/15)*ω*_*D*2_. Let us consider an example in which PC1 and PC2 have a thickness of 15*a*_2_ and 40*a*_2_, respectively, and we numerically calculate the incident-angle dependence of the transmission of the PC sandwich structure at the reduced frequency *δω* = *ω* − *ω*_*D*1_ = 0.0436*πc*/*a*_2_. The results are plotted by red open circles in Figure 3. The perfect transmission for the case of normal incidence (*θ* = 0) is a manifestation of “Klein tunneling”. At finite incident angles, we find some other transmission peaks with *T*(*θ*) = 1 at angles satisfying the relation *q*_1*x*_*D* = *mπ*  (*m* ∈ integers) as a consequence of Fabry-Perot resonances. When the incident angle is larger than the critical angle *θ*_*c*_ = sin^−1^((*V*_0_ − *δω*)/*δω*)≅41^0^, total reflection occurs and no transmission is found. The result of *T*(*θ*) from (21) for *δω* = 0.0436*πc*/*a*_2_ is also shown in Figure 3 (blue solid line). Excellent agreement is found between the prediction from (21) and the numerical result for the realistic structure. For comparison, we plot the transmission in Figure 3 for Dirac electrons in graphene (green dotted line) through a potential barrier with the same width *D* = 40*a*_2_. We take the energy *E* and potential height *U*_0_ as *E*/*ħv*_*F*_ = *δω*/*v*_*g*_ and *U*_0_/*ħv*_*F*_ = *V*_*o*_/*v*_*g*_, respectively, with *v*_*F*_ being the Fermi velocity of Dirac electrons. Note that Dirac electrons and pseudospin-1 EM waves have transmission peaks at the same angles due to Fabry-Perot effect. However, Dirac electrons with a pseudospin of 1/2 in graphene have much deeper transmission dips, which indicates that electrons experience much stronger anisotropic scattering by the 1D potential. It is found that the above difference is due to their different boundary conditions [47].

### 5.2. Supercollimation of Wave Packets in a Superlattice of Photonic Crystals

**Figure 4:**
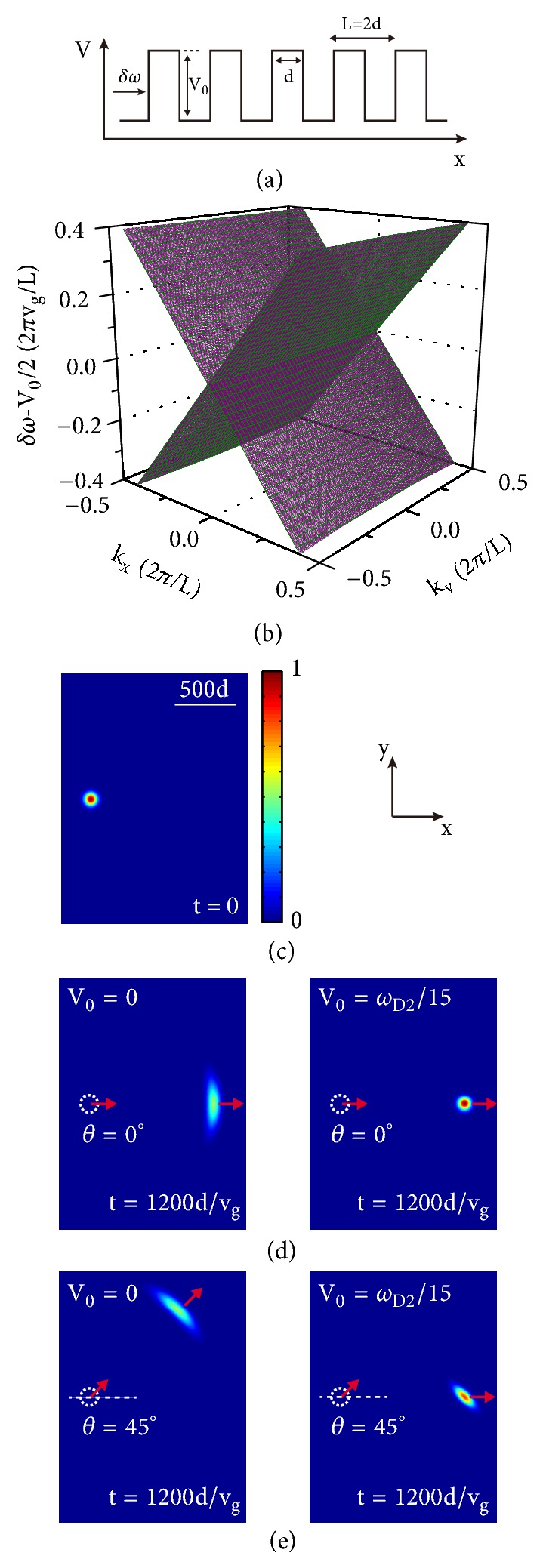
(a) Schematic picture of a pseudospin-1 superlattice formed by a Kronig-Penney type photonic potential. (b) The wedge-shaped dispersion structure of pseudospin-1 EM waves near *δω* = *V*_0_/2 for a superlattice realized by alternating stacks of PC1 and PC2 (see Figure 2). Both PC1 and PC2 have the same thickness *d* = 15*a*_2_, i.e., the spatial period of the superlattice is *L* = 30*a*_2_. The photonic potential is set at *V*_0_ = *ω*_*D*2_/15. (c) The magnitude of electric field distribution of a Gaussian wave packet at *t* = 0 with the reduced center frequency $\bar{\delta \omega_{c}}=0.06\pi v_{g}/L$ and the half width *r*_0_ = 30*d*. (d) and (e) The magnitude of electric field distributions of the Gaussian wave packet at *t* = 1200*d*/*v*_*g*_ in a single PC (left panel) and in the PC superlattice (right panel) with the initial propagation direction in an angle *θ* = 0^0^ (d) and 45^0^ (e), respectively. The results show the supercollimation induced by the superlattice. Adapted from [47].

The supercollimation here refers to a transport phenomenon that a wave packet can propagate a long distance in a 2D system while preserving its shape. Such a phenomenon was predicted theoretically in certain graphene superlattices consisting of two alternating layers with equal thickness due to anisotropic renormalization of the group velocity [13]. The occurrence of this phenomenon for pseudospin- 1/2 system requires a specific condition on the product of the barrier height difference and layer thickness. However, we will see that it is more robust for pseudospin-1 EM waves as collimation can occur without any condition [47]. We consider here a pseudospin-1 superlattice formed by a Kronig-Penney type of photonic potential along the* x*-direction [see Figure 4(a)]. The thickness of each layer is taken to be* d*, the potential height is *V*_0_, and lattice constant is* L*. For one unit cell of the periodic structure, the transfer matrix is the product of two parts,

(22) $$\begin{array}{c} M_{cell}=P\left(\;L-d\;\right)M\left(\;d\;\right), \end{array}$$

where **M**(*d*) is the part for the barrier layer as shown in (19) and **P**(*L* − *d*) describes the part of transfer matrix in the background layer,

(23) $$\begin{array}{c} P\left(\;L-d\;\right)=\left(\begin{array}{cc} e^{iq_{0x}\left(L-d\right)} & 0\\ 0 & e^{-iq_{0x}\left(L-d\right)} \end{array}\right). \end{array}$$

Using (22) in conjunction with the Bloch theorem, we obtain the following dispersion relation for the superlattice [47],

(24) cos⁡2kxd=cos⁡q1xdcos⁡q0xd−sin⁡q1xdsin⁡q0xd2δω−V0q0xδωq1x+δωq1xδω−V0q0x,

where *q*_0*x*_^2^ + *k*_*y*_^2^ = (*δω*/*v*_*g*_)^2^, *q*_1*x*_^2^ + *k*_*y*_^2^ = [(*δω* − *V*_0_)/*v*_*g*_]^2^, *k*_*x*_ is the Bloch wavevector, and *k*_*y*_ is the wavevector component along* y* direction. When *δω* = *V*_0_/2, it is found from (24) that the equifrequency curve is a straight line along the *k*_*y*_ axis with *k*_*x*_ = 0. It is clear that the group velocity in the* y* direction is zero for this equifrequency line. This result holds for all nonzero values of *V*_0_. We plot the dispersion relation of (24) in Figure 4(b) for a superlattice with *d* = 15*a*_2_, *L* = 30*a*_2_, and *V*_0_ = *ω*_*D*2_/15, where *a*_2_ is the lattice constant of PC2, and *V*_0_ is the potential shift between PC1 and PC2 (see Figure 2). A wedge structure can be clearly seen in Figure 4(b), exhibiting a dispersionless behavior along the *k*_*y*_ direction even when *δω* ≠ *V*_0_/2. A wedge equation, *δω* = *sv*_*g*_|*k*_*x*_| + *V*_0_/2 (*s* = ±1), can be found when |*k*_*y*_| < <*V*_0_/2*v*_*g*_ by expanding (24) in the vicinity of *δω* = *V*_0_/2. It can be seen clearly from this equation that within the wedge-like dispersion structure, the 1D periodic modulation of photonic potential reduces the group velocity in* y* direction to zero. On the other side, it makes no impact on the one in* x*-direction. Similar to the case in some special graphene (pseudospin-1/2) superlattice [12, 13], such wedge structure will lead to the supercollimation of pseudospin-1 wave packets in a superlattice of PCs due to the strong anisotropic renormalization of group velocity mentioned above, which means that a wave packet constructed in the frequency range of the wedge-like dispersion can be guided to propagate without distortion of shape along the potential modulation direction of the superlattice, independent of its initial direction of motion. It is worth noting here that for graphene, as a pseudospin-1/2 system, the wedge structure can only be achieved under certain special conditions that the periodic potentials *U*_0_ and the layer thickness* d* should satisfy the relation *U*_0_ = 2*πħv*_*F*_/*d* [12–14]. However, for a pseudospin-1 system, such a structure can be found for all nonzero values of *V*_0_ if each layer of the superlattice has the same thickness, i.e., *L* = 2*d* [47].

Figures 4(c)–4(e) show numerically the supercollimation phenomenon for a pseudospin-1 system in a Gaussian wave packet propagation simulation. In the simulation, we take the potential height as the photonic potential shift between PC1 and PC2 shown in Figure 2(b), i.e., *V*_0_ = *ω*_*D*2_/15, and the width *d* = 15*a*_2_. The superlattice is connected to a lead which is a PC with a photonic potential *V*_0_/2, and then a Gaussian wave packet is sent from the lead towards the superlattice. The initial wave packet in the form *E*_*z*_ = *E*_0_exp[−|**r** − **r**_**c**_|^2^/*r*_0_^2^ + *i ***k**_**c**_ · (**r** − **r**_**c**_)] is shown in Figure 4(c). Here **r**_**c**_ is the initial center of wave packet at a distance *d* away from the left end of the superlattice, $\bar{\delta \omega_{c}}=0.06\pi v_{g}/L$ is the reduced center frequency with the definition $\bar{\delta \omega_{c}}\equiv \delta \omega -V_{0}/2=v_{g}\left|k_{c}\right|$ and *r*_0_ = 30*d* is the half width of wave packet. Figures 4(d) and 4(e) show the evolution of the wave packet from the initial position for two cases: one with incident angle *θ* = 0^0^ [Figure 4(d)] and another with *θ* = 45^0^ [Figure 4(e)]. When the superlattice is absent, the wave packet propagates along the direction of initial center wave vector marked by the red arrow and spreads sideway rapidly. However, when the superlattice is present, the Gaussian wave packet always propagates along the potential modulation direction (*x*-direction), irrespective of the initial direction of motion. Furthermore, it is found that at the incident angle *θ* = 45^0^, the wave packet is stretched and tilted, which can be attributed to the strong dependence of the reflections on the value of *k*_*y*_ around *θ* = 45^0^ when the wave packet enters the superlattice from the lead. The wave packet will remain undistorted after it enters the superlattice [47].

### 5.3. Unconventional Anderson Localization in 1D Random Potentials

Wave travelling in a disordered medium can become localized due to interference effect. While the localization of scalar waves are well understood, the problem becomes more complex if the wave is propagating in a disordered medium with an underlying lattice structure. In the past few years, the effect of disorder on waves propagating in artificial materials has also received considerable attention [14, 64–76]. Many interesting phenomena have been predicted, such as suppression of Anderson localization in disordered metamaterials [67–72], angle-dependent electron transmission [74–76], and delocalization of relativistic Dirac particles in disordered 1D systems [73]. Here we review some surprising and counterintuitive wave localization behaviors for pseudospin-1 systems subjected to 1D disordered potentials.

In conventional 1D disordered materials, all states must become localized due to the coherent backscattering effect [77–81]. However, for pseudospin-1 systems, waves propagating in the normal direction experience no backscattering from a disordered 1D potential and only acquire a random phase in the spatial wave function [47]. Such one-way transport behavior was first discovered in pseudospin-1/2 systems [15, 73, 82, 83]. For pseudospin-1 EM waves considered in this review, the absence of backscattering in normal incidence can be understood from the classical wave impedance matching between any two neighboring layers, as we discussed in Section 4.2. Anderson localization can hence occur only for obliquely incident waves.

The systems considered here are 1D pseudospin-1 stacks of* N* uniform layers. Each layer is assumed to have a thickness* d*, but has a step-wise-constant random potential* V(x)*. Here we take the Dirac-like point of the background medium as the zero of the photonic potential, i.e.,* V* = 0. We consider the propagation of a plane wave through the multilayers at an oblique incident angle *θ* (*θ* ≠ 0) with a reduced frequency *δω*. The wave equation of pseudospin-1 EM waves in a 1D random potential can be written in a Hamiltonian form as (11),

(25) $$\begin{array}{c} H\psi =\left[\;v_{g}\vec{S}\cdot \vec{k}+V\left(\;x\;\right)I\;\right]\psi =\delta \omega \psi . \end{array}$$

For convenience, we use the normalized frequency $\overset{-}{E}=\delta \omega /v_{g}$ and random potential $\overset{-}{V}(x)=V(x)/v_{g}$ in the following. The normalized potential in each layer fluctuates independently, with a uniform distribution in the interval [$-\overset{-}{W}$, $\overset{-}{W}$], where $\overset{-}{W}$ is the random strength of the normalized potential. The localization length *ξ*, defined as the reciprocal of the Lyapunov exponent *γ*, can be obtained from the following relation,

(26) $$\begin{array}{c} \xi =\gamma^{-1}=-\underset{N\to \infty }{lim}\frac{2Nd}{{\langle lnT_{N}\rangle }_{c}}, \end{array}$$

where *T*_*N*_ is the transmission coefficient and 〈〉_*c*_ denotes ensemble averaging.

**Figure 5:**
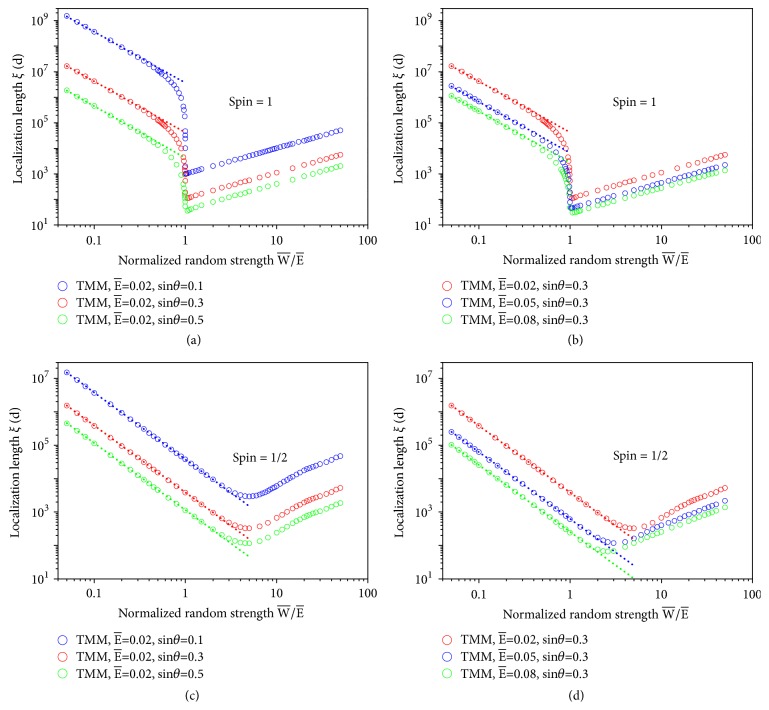
Localization length as a function of normalized random potential strength for different incident angles and $\overset{-}{E}$ in pseudospin-1 and -1/2 systems subjected to 1D disordered potentials. The results are calculated using the transfer-matrix method (TMM). (a) Localization length for three different incident angles in pseudospin-1 systems. (b) Localization length for three different values of $\overset{-}{E}$ in pseudospin-1 systems. (c) Same as (a), but for pseudospin-1/2. (d) Same as (b), but for pseudospin-1/2. The localization lengths at small $\overset{-}{W}$ are fitted by the dotted lines, showing a $\xi \propto {\overset{-}{W}}^{\;-2}$ behavior. Adapted from [52].

**Figure 6:**
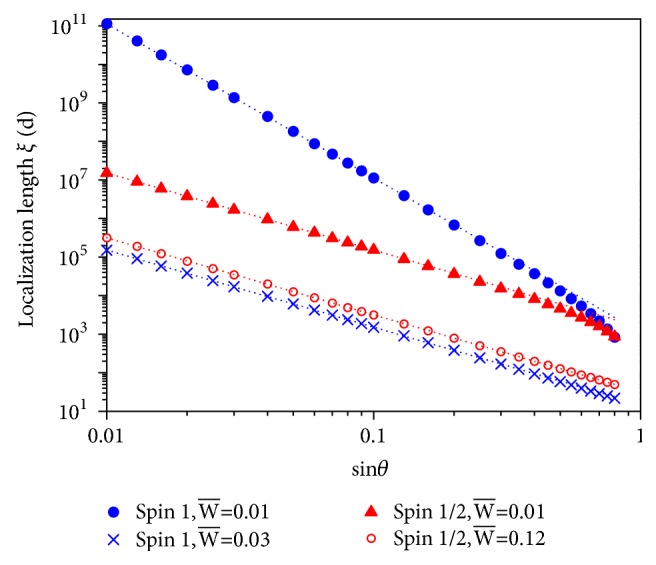
Localization length as a function of incident angle for $\overset{-}{E}=0.02$ and two random strengths in pseudospin-1 and -1/2 systems subjected to 1D disordered potentials. The two random strengths are chosen from the respective decreasing and increasing regions in Figures 5(a) and 5(c) for pseudospin-1 and -1/2 systems. For pseudospin-1 systems, in the case of $\overset{-}{W}=0.01$ ($<\overset{-}{E}$), the localization length at small *θ* (blue solid circles) is fitted by a dotted line, showing a *ξ* ∝ sin^−4^*θ* behavior. For all other three cases, the localization length at small *θ* is well fitted by *ξ* ∝ sin^−2^*θ*. Adapted from [52].

In Figure 5(a), we show the dependence of the localization length on the random strength $\overset{-}{W}$ at a particular normalized frequency $\overset{-}{E}=0.02$ for three different incident angles. Results are calculated by the transfer matrix method (TMM) and averaged over an ensemble of 4000 configurations. The sample size* N* is taken to be five times that of the localization length. We see that at small randomness, *ξ* decays with the randomness according to a general form $\xi \propto {\overset{-}{W}}^{\;-2}$ as expected. However, as $\overset{-}{W}$ increases, *ξ* drops suddenly to a minimum at a critical random strength ${\overset{-}{W}}_{c}=\overset{-}{E}$, regardless of incident angle, and rises immediately afterward. The sudden change of localization behavior near ${\overset{-}{W}}_{c}$ indicates the occurrence of some sharp transition between the two regions: $\overset{-}{W}<{\overset{-}{W}}_{c}$ and $\overset{-}{W}>{\overset{-}{W}}_{c}$. In Figure 6, we plot the localization length as a function of incident angle *θ* in the two regions. We find that the asymptotic *θ*-dependence of *ξ* indeed changes from *ξ* ∝ sin^−4^*θ* ($\overset{-}{W}<{\overset{-}{W}}_{c}$) to *ξ* ∝ sin^−2^*θ* ($\overset{-}{W}>{\overset{-}{W}}_{c}$) when $\overset{-}{W}$ moves across the critical random strength ${\overset{-}{W}}_{c}=\overset{-}{E}$. The V-shape turnaround of localization length occurring at a critical random strength and the change of the asymptotic behaviors in the *θ*-dependence are properties unique to pseudospin-1 systems and not found in any ordinary disordered systems. In ordinary materials, stronger disorder causes enhanced backscattering, and therefore a monotonic decrease of localization length with increasing disorder. In Figure 5(b), we show a similar plot as Figure 5(a) except that we now fix the incident angle at sin⁡*θ* = 0.3 and plot *ξ* as a function of random strength $\overset{-}{W}$ at three different values of $\overset{-}{E}$. It is clearly seen that the same localization characteristics are found in three different regions of $\overset{-}{W}$, i.e., $\overset{-}{W}<\overset{-}{E}$, $\overset{-}{W}\approx \overset{-}{E}$, and $\overset{-}{W}>\overset{-}{E}$.

For pseudospin-1/2 systems, the Hamiltonian can be written in the following form, $\left[\hbar v_{g}\vec{S}\cdot \vec{k}+V(x)I\right]\psi =E\psi$, where $\vec{S}$ is now a 2D Pauli vector in* xy* plane and* E* is the incident energy of pseudospin-1/2 quasiparticles. The localization length is plotted against the random strength for different incident angles and energies in Figures 5(c) and 5(d). Note here that for pseudospin-1/2 systems, we define the normalized energy $\overset{-}{E}$ and random potential $\overset{-}{V}(x)$ as $\overset{-}{E}=E/\hbar v_{g}$ and $\overset{-}{V}(x)=V(x)/\hbar v_{g}$, respectively. It is found that there is also a localization length minimum in pseudospin-1/2 systems after which the localization length increases again, but the sharp change in *ξ* found in pseudospin-1 systems is absent in pseudospin-1/2 transport. Instead, the localization length *ξ* crosses over smoothly from a decreasing behavior at small random strength to an increasing one at large random strength. The minimum occurs at the random strength around a few $\overset{-}{E}$. Furthermore, as can be seen in Figure 6, the *θ*-dependence of localization length in both decreasing and increasing regions shows an asymptotic behavior of *ξ* ∝ sin^−2^*θ*, in sharp contrast to pseudospin-1 behavior.

The above anomalous localization behaviors can be understood from the following analytic analysis. It turns out that (25) can be transformed into a scalar equation using a new coordinate* u* [52]:

(27) $$\begin{array}{c} \frac{d^{2}\Psi }{du^{2}}+\Psi =\frac{k_{y}^{2}}{{\left(\overset{-}{E}-\overset{-}{U}\left(u\right)\right)}^{2}}\Psi , \end{array}$$

where $u=\int_{0}^{x}\left(\overset{-}{E}-\overset{-}{V}\left(x^{\prime }\right)\right)dx^{\prime }$ and $\overset{-}{U}\left(u\right)=\overset{-}{V}\left(x\right)$. In (27), the oblique angle appears in the scattering term. In the case of normal incidence, i.e., *k*_*y*_ = 0, the scattering term vanishes, and (27) then describes wave propagation in a homogeneous medium and contains two general solutions, $\Psi \propto e^{\pm iu}=\exp\left[\pm i\int_{0}^{x}\left(\overset{-}{E}-\overset{-}{V}\left(x^{\prime }\right)\right)dx^{\prime }\right]$. We can see that the random phase accumulation due to 1D random potential during the one-way transport is now absorbed in the new coordinate* u*. Similarly, a scalar wave equation for pseudospin-1/2 systems can be constructed according to pseudospin-1/2 Hamiltonian equation,

(28) $$\begin{array}{c} \frac{d^{2}\Psi }{du^{2}}+\Psi =\frac{k_{y}^{2}}{{\left(\overset{-}{E}-\overset{-}{U}\left(u\right)\right)}^{2}}\Psi +k_{y}\Psi \overset{N+1}{\underset{i=1}{\sum }}U_{i}\delta \left(\;u-u_{i}\;\right), \end{array}$$

with $U_{i}=1/\left(\overset{-}{E}-{\overset{-}{v}}_{i}\right)-1/\left(\overset{-}{E}-{\overset{-}{v}}_{i-1}\right)$. Here *u*_*i*_ is the* i*th interface of the layered structure in the* u* coordinate and ${\overset{-}{v}}_{i}$ is the normalized random potential in* i*th layer.

We can qualitatively understand from the scattering terms in (27) and (28) why the *θ*-dependence of *ξ* in the two pseudospin systems behaves differently at small $\overset{-}{W}$. For ordinary disordered materials, the localization length in disordered 1D systems has the same order of magnitude as the mean free path, and the latter is inversely proportional to the square of the scattering strength [79]. For small values of *k*_*y*_, the *k*_*y*_^2^ dependence of the effective scattering potential in (27) leads to a *ξ* ∝ *k*_*y*_^−4^ (or sin^−4^*θ*) behavior, whereas in addition to the *k*_*y*_^2^ scattering term, (28) has another scattering term *k*_*y*_Ψ∑_*i*=1_^*N*+1^*U*_*i*_*δ*(*u* − *u*_*i*_) located at all* N*+1 interfaces. This interface term dominates at small *k*_*y*_ and lead to a *ξ* ∝ *k*_*y*_^2^ (or sin^−2^*θ*) behavior. The sudden drop of *ξ* near ${\overset{-}{W}}_{c}=\overset{-}{E}$ for pseudospin-1 systems can be understood from the diverging behavior of the scattering terms in (27) when $\left|\overset{-}{E}-\overset{-}{U}(u)|<|k_{y}\right|$ in some layers. In this case the waves become evanescent inside those layers. When $\overset{-}{W}$ exceeds the critical value ${\overset{-}{W}}_{c}=\overset{-}{E}$, the probability of having evanescent waves goes down as $\overset{-}{W}$ increases. Meanwhile, the scattering potentials in the propagating layers are weakened in general, as can be seen from the scattering terms in (27). Thus, the localization length goes up with increasing $\overset{-}{W}$. However, such a sudden drop of localization length is smeared out by the additional interface scattering terms in (28) so that a smooth crossover from the localization length decreasing behavior to an increasing one is found for pseudospin-1/2 systems.

Analytical solutions obtained from the surface Green function (SGF) method [52] show that for pseudospin-1 systems, when $\overset{-}{W}>\overset{-}{E}$, the evanescent waves emerge and contribute to *γ* a sin^2^*θ* term, which dominates over the sin^4^*θ* term from propagating waves, resulting in the transition of the *θ*-dependence shown in Figure 6. For pseudospin-1/2 systems, both evanescent and propagating waves give a sin^2^*θ* dependence in *γ*, leading to the same asymptotic *θ*-dependence for all $\overset{-}{W}s$ [52].

The 2D transport properties of pseudospin-1 waves also have unique features. The scattering of pseudospin-1 waves from a circularly symmetric potential barrier has been studied [53, 54]. For the case of 2D PCs, such a potential barrier can be realized by changing the length scale in a selected circular domain. Many interesting scattering properties of pseudospin-1 waves from such a circular potential barrier have been predicted theoretically, including perfect caustics, revival resonance, isotropic transport, and superscattering [53, 54].

## 6. Conclusions

We have reviewed the realization of pseudospin-1 physics in classical wave systems using 2D dielectric photonic crystals possessing Dirac-like dispersions at** k**=0. The physics of those systems near the Dirac-like point frequency can be described by an effective pseudospin-1 Hamiltonian which has three degrees of freedom, representing the projection of the real space wave function to monopole and dipole excitations. A photonic potential which can shift the Dirac-like cone rigidly can be realized simply by changing the length scale of the dielectric PCs. Due to the unique Dirac-like conical dispersion, waves in pseudospin-1 systems interact with external potentials in unusual ways, leading to many exotic transport properties. We have also reviewed a number of unusual pseudospin-1 wave transport phenomena, including super Klein tunneling, supercollimation, and unconventional Anderson localization. Without the requirements of extremely low temperature and atom manipulation, the fabrication of photonic systems is much easier than that for ultracold-atom and electronic systems. With the recent rapid progress of modern microelectronic technologies, one can now achieve long enough coherent length to form complex junctions, especially in the microwave regime. Also, the absence of complex interactions between photons facilitates the advances of theories and makes the experimental observations and characterizations much easier. With the rapid development of the field of pseudospin-1 physics, there emerge lots of interesting phenomena, such as the superscattering of pseudospin-1 waves from a circular potential barrier [54], perfect caustics inside a circular barrier [53], current enhancement induced by the flat band in nonequilibrium transport of pseudospin-1 particles [55], and nonuniversal long-wavelength critical behavior of Anderson localization [51]. We expect that photonic crystals can be a good platform to test experimentally these theoretical predictions of pseudospin-1 physics. The demonstration of the above features of pseudospin-1 systems may provide us novel ways in controlling the propagation of pseudospin-1 particles, leading to possible applications in optical circuits and optical imaging beyond the diffraction limit.
